# Social media sites users' choice between utilitarian and informational reinforcers assessed using temporal discounting

**DOI:** 10.3389/fpubh.2023.960321

**Published:** 2023-02-10

**Authors:** Oscar Robayo-Pinzon, Sandra Rojas-Berrío, Mario R. Paredes, Gordon R. Foxall

**Affiliations:** ^1^School of Business Administration, Universidad del Rosario, Bogotá, Colombia; ^2^Faculty of Economics, Universidad Nacional de Colombia, Bogotá, Colombia; ^3^Cardiff Business School, Cardiff University, Cardiff, United Kingdom; ^4^School of Management, Reykjavik University, Reykjavik, Iceland

**Keywords:** behavioral economics, temporal discounting, utilitarian reinforcement, informational reinforcement, social media

## Abstract

**Objective:**

This study provides a first approach to the use of the Multiple-Choice Procedure in social media networks use, as well as empirical evidence for the application of the Behavioral Perspective Model to digital consumption behavior in young users in conjunction with a methodology based on behavioral economics.

**Participants/methods:**

The participants were part of a large university in Bogotá, Colombia, and they received an academic credit once they completed the online questionnaire. A total of 311 participants completed the experiment. Of the participants, 49% were men with a mean age of 20.6 years (SD = 3.10, Range = 15–30); 51% were women with a mean age of 20.2 years (SD = 2.84, Range = 15–29).

**Results:**

Among the total participants, 40% reported that they used social networks between 1 and 2 h a day, 38% between 2 and 3 h, 16% for 4 h or more, and the remaining 9% used them for 1 h or less per day. The factorial analysis of variance (ANOVA) allowed us to identify a statistically significant effect of the delay of the alternative reinforcer, that is, the average crossover points were higher when the monetary reinforcer was delayed 1 week, compared to the immediate delivery of the monetary reinforcer. There was no statistically significant effect of the interaction between the magnitude of the reinforcer and the delay time of the alternative reinforcer.

**Conclusions:**

This study supports the relative reinforcing value of an informational reinforcement consequence such as social media use, which is sensitive to both the magnitude of reinforcement and the delay in delivery as individual factors. The findings on reinforcer magnitude and delay effects are consistent with previous research that have applied behavioral economics to the study of non-substance-related addictions.

## 1. Introduction

Social media networks (SMN) use is one of the most popular online activities. It has been defined as “any online resource that is designed to facilitate engagement between individuals” (Bishop (1), p. 63). Its growth has been exponential worldwide (2). By January 2022, there were more than 4.62 billion active social media users around the world −58.4% of the world's population. In addition, social media sites were the top type of website visited by global Internet users. The average daily time a user spent on a social media platform was 2 h and 27 mins (3). Among the most popular social media sites were Facebook—with over 2.9 billion users—followed by YouTube with 2.2 billion, and Instagram—with 2 billion (4). In terms of the accelerated adoption and growing use of social networks, young people make up one of the most frequent user groups on social media platforms. To illustrate, in the U.S, around 89% of teenagers between the ages of 13 and 17 have access to a smartphone, and they report constant use of social media sites (5).

The literature has identified self-expression, community building, entertainment, and emotional support as among the major motives for social media use (6). Furthermore, teenagers are one of the most vulnerable populations to develop adverse effects from extensive social media usage (7–9). Research suggests that negative effects on health and wellbeing—including life dissatisfaction (10, 11), sleep deprivation (12), eating disorders and body image concerns (13)—can be derived from prolonged use of social media. As well, there can be negative effects on students' academic performance (14).

Recently, behavioral economics, a discipline that combines operant psychology and microeconomics, has been employed to address the problematic use of technologies such as smartphones or Internet use (15, 16). In particular, applying the concept of temporal discounting to the study of the phenomenon, tries to establish relationships between dependence and time of use and a tendency toward impulsivity, measured through scales or intertemporal choice task procedures (16–18).

Behavioral economics provides a framework for the analysis of choice behavior and decision-making by describing individual allocation of behaviors between simultaneous available potential reinforcers (19). Specifically, the Multiple Choice Procedure (MCP) is a laboratory procedure developed to investigate the relationship between drug use and alternative reinforcers, providing an index of relative reinforcing value for that substance (20). This tool is based on the principle that the relative reinforcing value of substance use depends on a set of variables that correspond to both the inherent properties of the substance and the physical and social environment (21). These variables include primarily, the magnitude of the substance that is available for use and the number of alternative reinforcers found in the environment (22). It has been an effective approach to researching addictive behaviors that elicit public health concerns, such as video game playing (23), gambling (24), and substance consumption such as marijuana (25), alcohol (26), cocaine (27), and heroin (28).

The MCP uses a structured questionnaire to assess individuals' decisions by providing them with two or more choices between a motive of interest (e.g., drugs) and an increasing value of alternative reinforcers (e.g., actual or fictional monetary payments), until the crossover point is reached, which is the stage when the participant no longer chooses the stimulus of interest and begins to choose the alternative reinforcer (20). Therefore, the crossover point is defined as the magnitude at which the participant begins to choose the alternative reinforcer (e.g., money) over the target substance or behavior (e.g., social media networks use). A higher value of the crossover point represents a higher relative value of reinforcement for the target substance or behavior, that is, if the participant chooses to change his or her preference to a higher amount of monetary reward, this indicates that the relative value of the substance is higher (29, 30). This method is based on the concept of delay discounting, which refers to the devaluation of later results of behavior. That is, as the outcome moves further into the future, it has less influence over the current choice (31). Discounting delayed reinforcers denotes that individuals have to choose between the value of a delayed reinforcer that is discounted, compared to the value of an immediate reinforcer (32, 33). These decisions that are made in the face of consequences that are available at different points in time are known as intertemporal choices and are sensitive to the magnitudes of the rewards associated with the alternatives involved in the choice. Specifically, consumers can be expected to choose to wait for larger rewards than for smaller rewards, a phenomenon known as the magnitude effect (34–39). The implication is that individuals have to choose between a small sooner reward (SS) and a larger outcome available later (LL) (40). For instance, individuals may save up for an appealing product (LL) or impulsively buy something (SS), or they may invest money for the future (LL) or to spend that money to get the last smartphone (SS). Typically, most people would prefer SS to LL (41).

Furthermore, consumer behavior literature—specifically the Behavioral Perspective Model (BPM) (42, 43)—states that behaviors are affected by two kinds of consequences (utilitarian and informational) that may serve as reinforcements or punishments. Utilitarian reinforcement (UR) is that associated with the direct use or consumption of the product, whose benefits (or lack thereof) act as reinforces or punishers, influencing the purchase intention. On the other hand, informational reinforcement (IR) relates to the symbolic and social effects derived from the purchase or consumption process, which are given in the specific context of the customer (44). Moreover, these decisions are affected by temporal considerations. Many routine purchasing decisions do not involve major discrepancies as to whether it is desirable to buy or consume now or to wait for some time before having the opportunity to consume a product. This would be the case for most fast-moving consumer goods. For these products, waiting a long time may even be counterproductive, as there would be no more of a benefit in the future than at present, and prices may even increase in the future. However, some products can provide a very pleasurable consequence in the short term, leading to a pattern of choice that can lead to dependence or addiction such as with alcoholism and cigarette smoking (45). In the case of the use of social media networks, it is possible that since it is a behavior with such a high frequency of daily occurrence with brief exposures (micro-moments of use), the user does not perceive the existence of a conflict between the immediate pleasure of this informational reinforcer and a long-term goal, such as having better grades at the end of the term or better job performance indicators at the end of the year. On the other hand, monetary rewards (real or fictitious) have been used as an alternative reinforcer (23, 24, 46), and although they could be considered both utilitarian and informational reinforcers, due to the potential to generate prestige and admiration in others, in these experimental conditions the amounts used could hardly be associated with such a social acceptance effect, nor would it be easy for participants to make this visible to their peers.

Few studies seek to explore alternatives for reducing dependence or time spent on digital platforms based on these principles. Although studies have recently been published that apply the concepts of utilitarian and informational reinforcement in behavior analysis-based therapy contexts, smartphone and social network dependence behaviors have not yet been taken into account. Therefore, the present study aims to contribute to the growing literature on IR in economic research (47, 48). Extant research has primarily focused on the exploration of smartphone dependence (16, 49, 50). However, there is evidence that there are differences among smartphone devices addiction and social network services addiction (51). Also, limited research has explored how to establish the relative reinforcement value of a monetary reward over the option to use social media networks. No prior study has attempted to apply the MCP to establish these relative reinforcement values between monetary rewards, real or fictional, and social media networks use over different periods of time. Therefore, this study intends to answer the following research question (RQ): What is the relative reinforcement value of monetary rewards vs. different time periods of social media networks use?

To accomplish this objective, this research applies an experimental design based on contingency management (52), in which it was established whether a utilitarian reinforcement (monetary reward) is preferred to an informational one (social media sites usage) in different conditions of delivery time of the reinforcers.

## 2. Materials and methods

### 2.1. Participants

The participants were part of a large university in Bogotá, Colombia, and received an incentive of hours of academic credit once they completed the online questionnaire. A total of 390 participants completed the experiment, of whom 44 were excluded because they indicated multiple crossover points in the MCP (despite instructions to cross only once) or because data were missing in at least one version of the MCP. Additionally, 35 participants were excluded due to their age. The inclusion criterion in this statistical analysis was that the participants were young adult students between the ages of 18 and 30 years. The remaining 311 participants were 49% male with a mean age of 20.6 years (SD = 3.10, Range = 15–30) and 51% female with a mean age of 20.2 years (SD = 2.84, Range = 15–29); 87% were graduates of an undergraduate degree program or were currently students in one; 36.7% reside in households with a medium socioeconomic level, 35.4% were in low-middle stratum, 13.5% were in low stratum, 11.3% were in a high-middle stratum; 2.3% were in a high stratum, and the remaining 1% was in the very lowest stratum.

### 2.2. Instruments

#### 2.2.1. Multiple choice procedure

Participants were asked to make hypothetical discrete decisions between fixed magnitudes of reinforcement (social media networks time usage) and increasing amounts of a monetary reinforcement (Colombian pesos) (see Appendix in Supplementary material). Six versions of the questionnaire were designed, which is consistent with previous studies that have used the MCP, in which an amount of time or money (e.g., money for gambling or time for playing video games) was combined with increasing magnitudes of alternative monetary reinforcement (20, 23, 24). For each version of the MCP participants were instructed that once they selected money, they would have to continue selecting money for that version of the MCP. Therefore, the crossover point for each version corresponds to the monetary value at which the change in preference from using social media networks to receiving the respective amount of money occurred. After receiving the respective instructions to carry out this laboratory study, they answered the six versions of the MCP, including three different magnitudes of informational reinforcement (that is, 5, 15, and 45 mins of social media network use) and two different time points to receive the alternative monetary booster (i.e., immediately or delayed by 1 week). The social media network usage times were selected by the researchers from a pilot test. Each magnitude of the first booster was paired with the two-time points to receive the monetary booster and presented in sequential order (i.e., 5 mins vs. money right away, 5 mins vs. money a week later, 15 mins vs. money immediately, 15 mins vs. money 1 week later, 45 mins vs. money right away, 45 mins vs. money 1 week later). The monetary booster started at $10,000 (about 2.5 dollars), increased to $12,000 (about 3 dollars), then to $15,000 (about 4 dollars), then to $20,000 (about 5.2 dollars), and finally to $25,000 (about 6.5 dollars). These values correspond to Colombian pesos. Each participant responded to 30 discrete choice situations, corresponding to five choices for each of the six versions of the MCP. Each of these versions resulted in a single crossover point, which is the data of interest in this measurement procedure and was conceptualized as the relative reinforcement value of using social media networks (informational) compared to an alternative monetary reinforcement (utilitarian).

#### 2.2.2. Social Media Addiction Scale

The Social Media Addiction Scale (SMAS) was originally developed by Tutgun-ünal et al. (53), shows adequate levels of validity and reliability, and has 41 items covering four dimensions: “occupation,” i.e., how much time the participant thinks he/she is busy checking his/her social media networks; “mood modification,” i.e., how often the user takes refuge in social media to escape reality or the daily routine; “relapse,” the user's unsuccessful efforts to control the amount of time spent checking social media networks; and the “conflict” between the intention to use social networks more at times when there are other tasks or activities to be done, which results in a contradiction that generates discomfort in the user. The SMAS consists of a five-point Likert scale [(1) Never, (2) Rarely, (3) Sometimes, (4) Often, and (5) Always]. The scale was initially back translated with a subsequent cognitive validation pilot test. The result was the final Spanish version (see Appendix in Supplementary material). Analyses were conducted using total SMAS scores, which were obtained from the sum of the scores for each item.

#### 2.2.3. Procedure

Given that all participants responded to all six versions of the MCP as well as to the questions on SMN use and the SMAS scale, and in line with previous studies (20, 23, 24), random assignment to experimental or control groups was not required. The participants completed an online questionnaire *via* the Lime Survey platform. This was composed of a section of demographic and social media usage questions, the six versions of the MCP, and the SMAS. All participants signed informed consent waivers, and the method was approved by the university's institutional ethics committee.

## 3. Results

Forty percent of the participants reported that they use social networks between 1 and 2 h a day; 38% between 2 and 3 h; 16% for 4 h or more; and the remaining 9% use them for 1 h or less per day. Table 1 shows the means and standard deviations for the full sample and by gender, for each of the main variables. Most the participants reported that the social media network they use the most is Instagram (53.3%), followed by TikTok (20.6%) and Facebook (13.3%). The results in the SMAS indicate that on average the participants have a low level of dependence on the use of social networks (M = 91.94, SD = 27.20, Range = 41–181), it is noteworthy that 75% of the participants present a low level of dependence on the use of social networks and in very few cases they present a high level of dependence. As can be seen in Table 1, there is no significant difference in the crossover points of the six versions of the MCP, nor is there a significant difference in the SMAS according to gender.

**Table 1:** Means and standard deviations for the main study variables.

**Variable**	**Full sample (*****N*** = **311)**		**Males (*****n*** = **152)**		**Females (*****n*** =**159)**		
	***M***	**SD**	***M***	**SD**	***M***	**SD**	***T*** **value**
MCP5i	10,549.84	2,516.38	10,761.59	2,777.79	10,356.69	2,253.24	0.17
MCP15i	11,553.05	5,137.11	11,721.85	5,178.36	11,420.38	5,154.20	0.65
MCP45i	11,157.56	3,691.00	11,165.56	3,921.62	11,171.97	3,501.24	0.94
MCP5w	11,922.83	5,581.12	11,788.08	5,823.64	12,089.17	5,401.06	0.60
MCP15w	11,832.80	4,874.33	12,218.54	5,598.68	11,496.82	4,092.87	0.22
MCP45w	12,286.17	6,463.68	11,907.28	7,031.69	12,694.27	5,922.21	0.26
SMAS	91.94	27.20	91.78	27.08	92.10	27.17	0.92

*N* = 311. MCP5, MCP version with 5 min of social media networks (SMN) use; MCP15, MCP version with 15 min of SMN use; MCP45, MCP version with 45 min of SMN use; i, immediate reinforcement; w, 1 week delay of reinforcement, i.e., MCP15i, MCP version with 15 min of SMN use and an immediate monetary reinforcement. SMAS: Social Media Addiction Scale.

**Figure 1:**
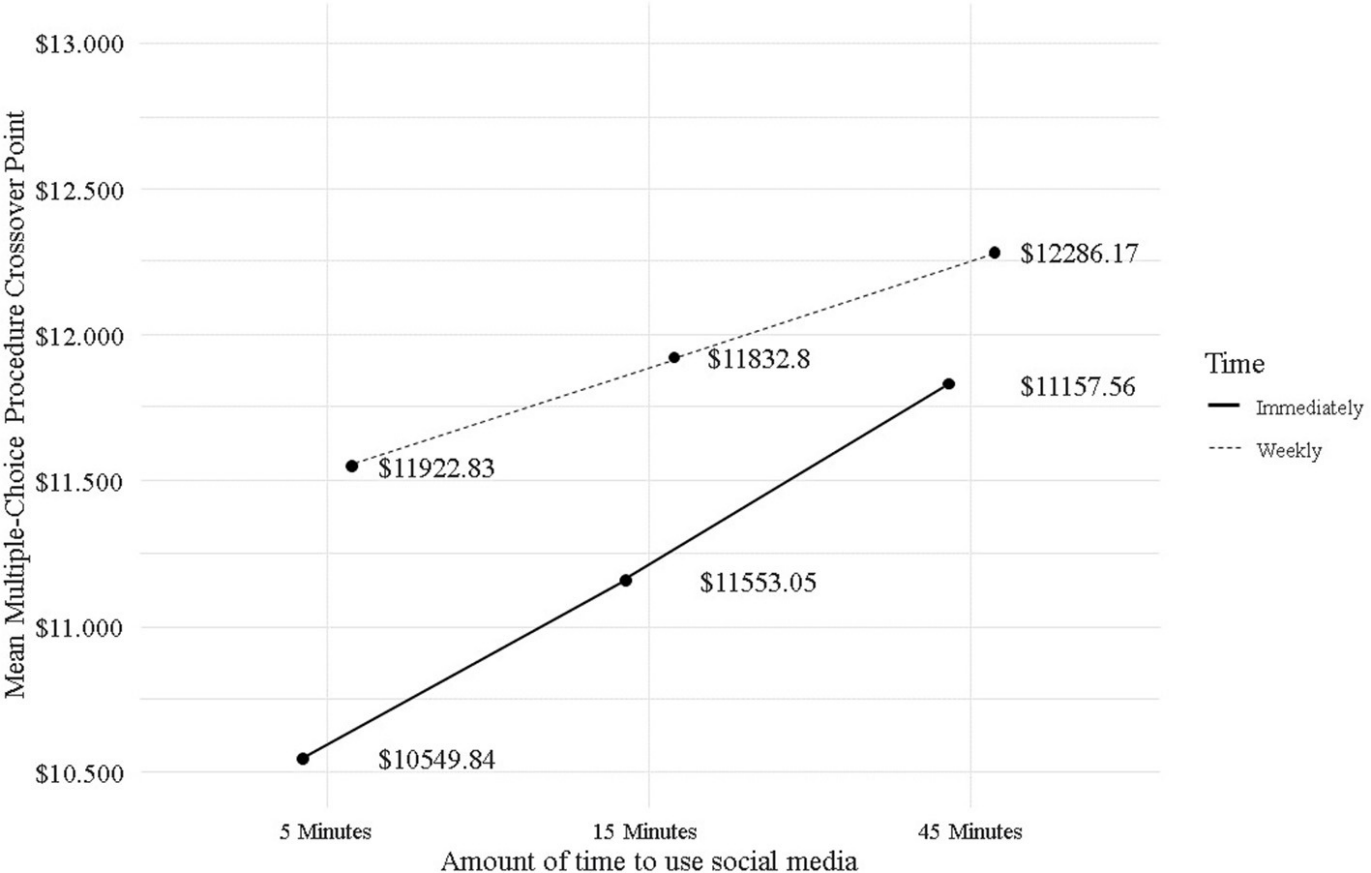
Mean Multiple Choice Procedure crossover points of each of the six versions.

To test whether the MCP evaluating hypothetical choices between social media use and an alternative monetary reward was sensitive to the magnitude of the reward and/or delay, a 2 × 3 repeated measures factorial ANOVA [Delay (immediate, 1-week delay) Magnitude (5, 15, 45 mins to use social media)] was conducted, using MCP crossover points as the dependent variable. For the analysis, it was verified that the responses had a normal distribution and that there were no significant differences in variance. The separate models for men and women yielded non-significant results. In the same way, the models separated by social media network yielded non-significant results. Therefore, only the model for the complete sample is presented. The average values of the crossover points for the six versions of MCP are presented in Table 1 and Figure 1.

The factorial ANOVA (see Table 2) allows us to determine that there is a statistically significant effect of the delay of the alternative reinforcer [*F*_(1, 310)_ = 10.21, *p* < 0.05]. That is, the average crossover points were higher when the monetary reinforcer was delayed 1 week (M = 11,921) compared to the immediate delivery of the monetary reinforcer (M = 11,180). This indicates that the relative reinforcement value of using social media networks increases when the delivery of the monetary reinforcer is delayed. It is observed that there is no statistically significant effect of the interaction between the magnitude of the reinforcer and the delay time of the alternative reinforcer. Additionally, there is a statistically significant effect of the magnitude of the reinforcer [*F*_(2, 620)_ = 9.5, *p* < 0.05], such that the average crossover points were higher for 45 mins (M = 12,059) of time to use social networks, followed by 15 mins (M = 11,540) and then 5 mins (M = 12,059), demonstrating a temporal magnitude effect for the relative reinforcement value of using social media networks.

**Table 2:** Factorial analysis of variance for repeated measures.

**Effect**	**DFn**	**DFd**	***F***	***p < * 0.05**	**ges**
1. Magnitude	2.00	620.00	9.50	0.00^*^	0.01
2. Time	1.00	310.00	10.21	0.00^*^	0.01
3. Magnitude: Time	2.00	620.00	1.25	0.29	0.00

* Statistically significant *p*-value.

**Figure 2:**
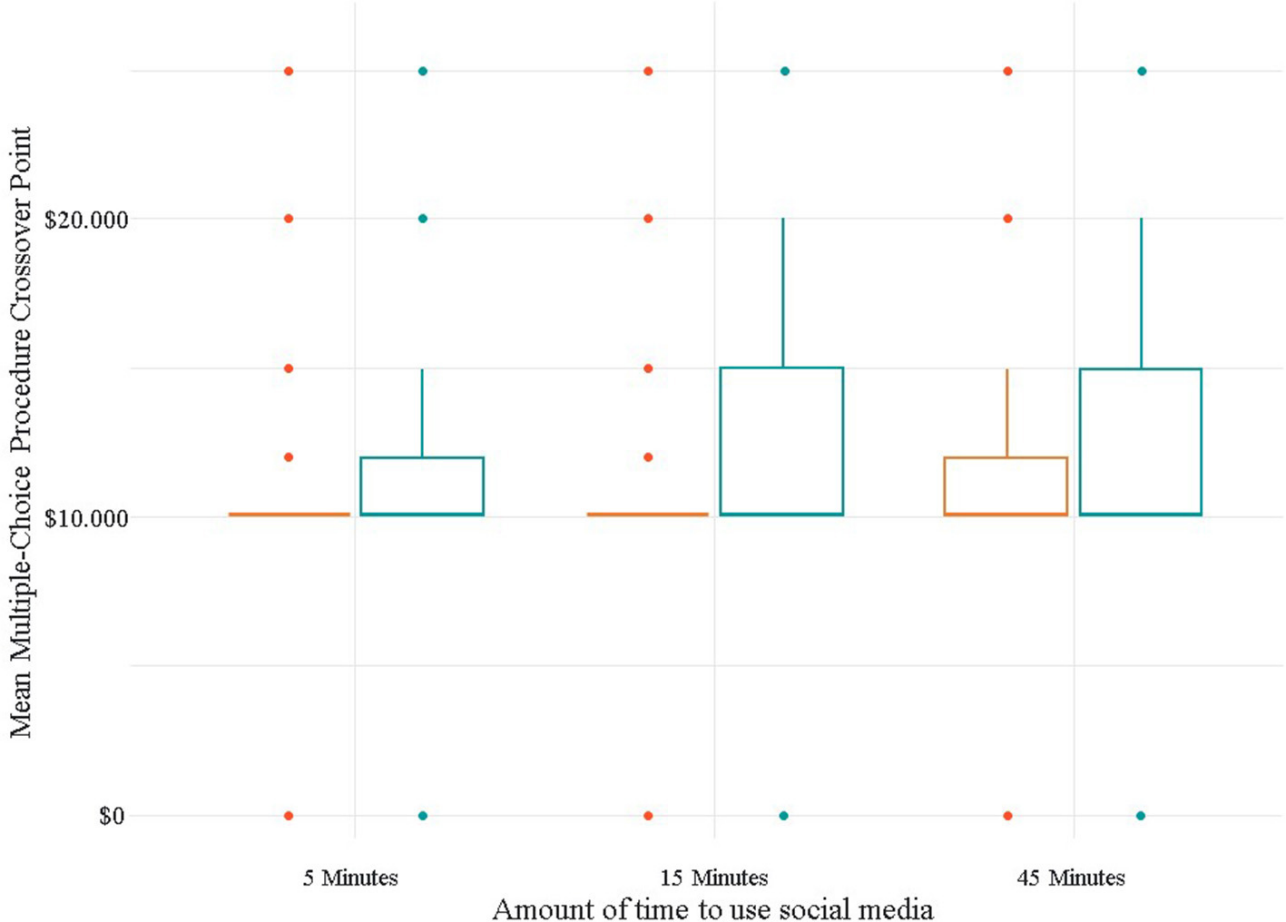
Differences in the average crossover points according to the magnitude of the reinforcer.

Because the interaction is not significant, the main effects of each of the two variables (the magnitude of the reinforcer and the delay time of the alternative reinforcer) must be interpreted. A significant main effect can be followed with pairwise comparisons. A Student t-test with a Bonferroni correction detected that there are significant differences between the average crossover points between the magnitude of the reinforcer of 5 mins of use of social networks and 15 mins (*t* = 3.27, *p* < 0.05), and also between the mean crossover points between the reinforcer magnitude of 15 mins of social media use and 45 mins (*t* = 4.40, *p* < 0.05). These results are illustrated in Figure 2.

However, as shown in Table 3, the bivariate correlations between the crossover points of each version of the MCP (the mean crossover point of the MCP and the total scores of the SMAS) do not present a significant statistical correlation. Therefore, it is not feasible to perform a multiple hierarchical regression analysis to assess the contribution of the MCP to the prediction of the total SMAS score. Bearing in mind that the Social Media Survey (SMS) variable is a categorical variable that measures the amount of time in hours that participants usually dedicate daily to social networks, an ANOVA is performed to identify whether the total score of the SMAS is seen as significantly modified, depending on the amount of daily use. The results are shown in Table 4.

**Table 3:** Bivariate correlations.

**MCP1**	**MCP2**	**MCP3**	**MCP4**	**MCP5**	**MCP6**	**MCP_PROM**	**SMDS**
1	0.225	0.502	0.128	0.241	0.216	0.444	0.075
0.225	1	0.274	0.703	0.299	0.407	0.742	0.093
0.502	0.274	1	0.356	0.306	0.207	0.563	0.077
0.128	0.703	0.356	1	0.388	0.493	0.801	0.091
0.241	0.299	0.306	0.388	1	0.546	0.697	0.003
0.216	0.407	0.207	0.493	0.546	1	0.77	0.077
0.444	0.742	0.563	0.801	0.697	0.77	1	0.099
0.075	0.093	0.077	0.091	0.003	0.077	0.099	1

**Table 4:** ANOVA between SMAS scores and time of daily use of social media networks.

**Df**	**Sum Sq**	**Mean Sq**	***F* value**	**Pr(>*F)***
3	18,742.1	6,247.38	10.5844	0.00
302	178,254	590.246		

**Figure 3:**
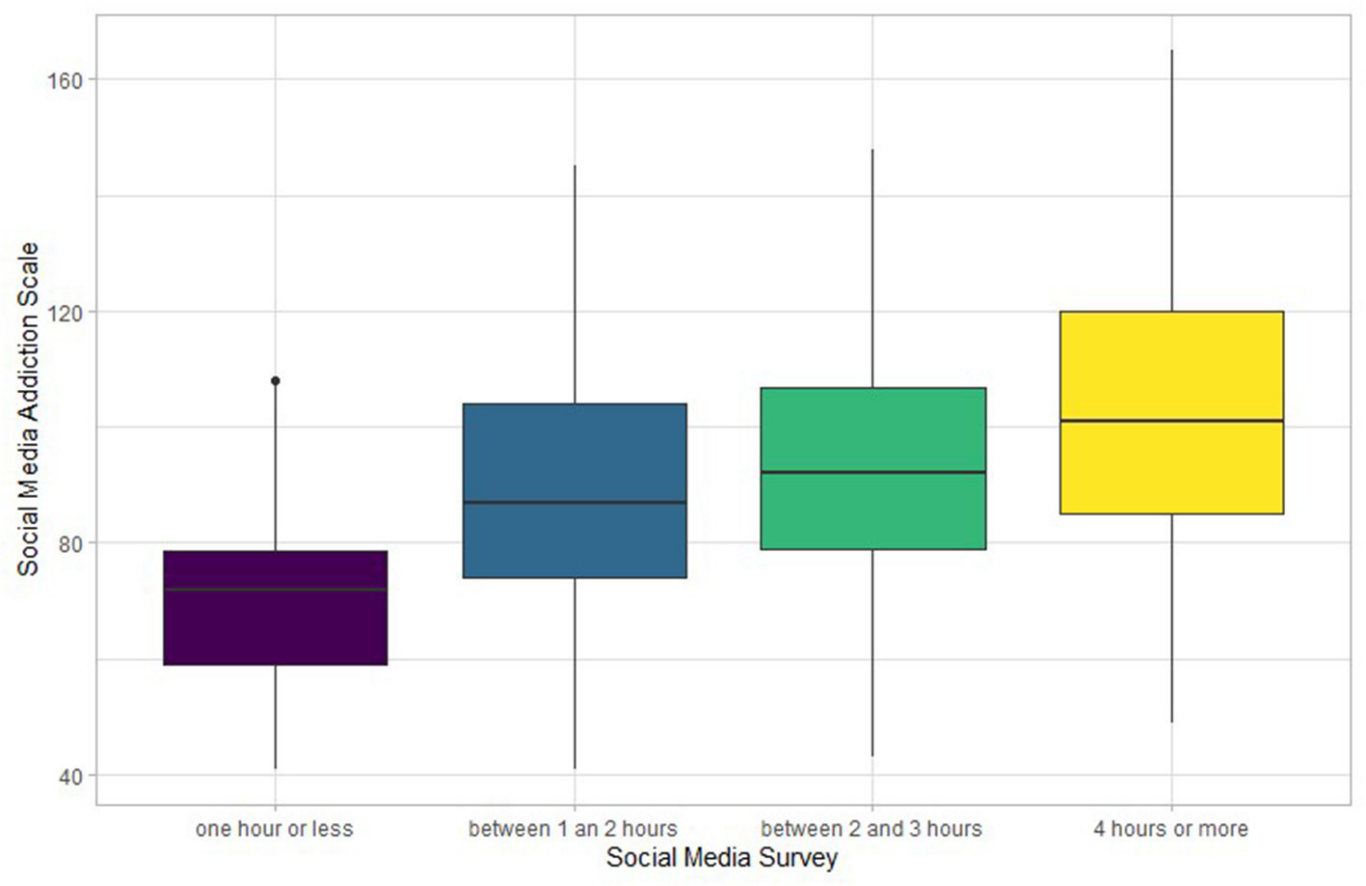
LSD Test—Social Media Addiction Scale in function of Social Media Survey.

The analysis of variance (ANOVA) demonstrates that there is a statistically significant effect on the amount of time that the participants use each day [*F*_(3, 302)_ = 10.58, *p* < 0.05], that is, the SMAS score was higher when the usage time was 4 h or more, compared to the other amounts of time. For this result to be valid, the assumptions of the ANOVA must be satisfied. Therefore, the Shapiro Wilk test was performed (*p* > 0.05), which indicates that the assumption of normality was met. The Bartlett test was also applied (*p* > 0.05), which indicates that the assumption of homogeneity of variances is fulfilled. The average total SMAS score for participants who reported the use of 1 h or less of social network time was 70.5 (SD = 18.8). For participants who reported the use of between 1 and 2 h (the majority of the participants), the average total was 89.1 (SD = 24.3). For participants who reported between 2 and 3 h use, it was 91.9 (SD = 24.3). Finally, for participants who reported the use of 4 h or more it was 103. That is, as the amount of time of use increases, there is also an increase in the total SMAS score, which is illustrated in Figure 3.

This conclusion was confirmed by applying the least significant difference (LSD) test, which allows us to conclude that there are statistically significant differences between each pair of categories of the SMS, except for the combination: “Between 1 and 2 h” and “Between 2 and 3 h.”

## 4. Discussion

This study intended to answer the RQ: What is the relative reinforcement value of monetary rewards vs. different time periods of social media networks use? a relationship unexplored by previous literature. Overall, the results suggest that there is an effect of both delay and magnitude. In the case of the delay effect, it was found that the average crossover point, or the stage when participant no longer choose the social media networks use and preferred the monetary reward, was higher when the monetary reinforcer was delayed 1 week, compared to the immediate delivery of the monetary reinforcer. This means that participants expect a higher amount of monetary reward for having to wait a week, thus representing a higher discount rate for the 1-week waiting interval (54). On the other hand, for the magnitude effect, it was found that participants preferred alternatives with a higher monetary reward as the time spent using social media networks increased. That is, the average crossover point was higher for the condition of 45 mins (compared to the 15- and 5-mins conditions). This implies that the discount rate is higher for smaller magnitudes (5 and 15 mins) compared to a larger magnitude (45 mins). Taken together, these findings indicate that the relative reinforcement value of using social media networks increases when the delivery of the monetary reinforcer is delayed and the time available to use social networks is higher.

Results of this study contribute to the literature on non-substance dependence by employing the MCP as an emergent methodology in applying behavioral economics, specifically temporal discounting, in the context of social media networks usage. Similar to previous studies in other contexts, the MCP was found to be a valid method for estimating the relative reinforcement value of social media network use as an informational reinforcement alternative to a utilitarian alternative, such as a monetary reward. However, there are differences between the types of behaviors reported in the literature and the use of social media networks. Social media use may be seen as a collection of micro moments of consumption.

This feature constitutes a main difference with similar behaviors such as gambling or videogames. Social media networks are permanently available for users through the smartphone. The personal character and physical presence of the smartphone provides the permanent access, therefore, to the informational reinforcement in lower but frequent “doses.” From the results obtained, it can be observed how the relative value of the alternative utilitarian reinforcer increases as it grows in magnitude, especially when the delay is 1 week, in order to modify the preference that participants show for the use of social networks as an informational reinforcer, and thus reverse the continuum of choices in favor of this immediate and pleasant option. Although in the literature on temporal discounting it has been reported that its absence or low levels are associated with routine purchases, usually of food products (44), it could be considered from the evidence found in this study, which is consistent with previous studies, that the use of social networks, being an informational reinforcer, would be presented as a routine pattern of choice with a high frequency of daily occurrence, something that is noteworthy and relevant to confirm in future research.

It should also be considered that in Colombia the use of social networks is among the highest in the world, with 81.3% of the population using social networks by January 2022, showing a year-on-year growth of 7.2%, i.e., 2.8 million new users per year (55). In this context, the immediate accessibility to the informational reinforcement and with almost 2.5 h of average daily social media usage globally (56), makes this behavior different to similar activities such as gambling or playing video games. Therefore, more research is needed in the field.

On the other hand, this study supports the relative reinforcing value of an informational consequence such as social media use and its sensitivity to both the magnitude of reinforcement and the delay in delivery as individual factors. However, there was no significant effect on the crossover points for the interaction of the categories of these two variables. As suggested above, it is possible that this behavior is qualitatively different from gambling or playing video games, given the fragmented nature of usage throughout the day. This makes it difficult for users to subjectively estimate how much time they spend using social networks per day. Another difference that should be considered when interpreting the results is that fewer versions of the MCP were used in this study compared to previous studies. This may have resulted in lower sensitivity for the measurement of crossover points because the differences in the magnitudes of the monetary reinforcers were larger between each discrete choice situation. However, the significant effect of magnitude and delay indicates that as the amount of time to use social networks increases, the value of the monetary reward for the crossover points also increases, and these values are higher for the One Week Delay condition. This result is consistent with what has been reported in previous studies (23). The relative reinforcement value of social media networks use increases the longer the time of use, with the effect being greater for the One Week Delay condition.

Further analysis established that there is a significant association between the categories of daily social networking time (SMS) and the SMAS score, a result similar to that reported in previous studies. In this case it was found that the average SMAS score was significantly higher for participants who reported a daily use of 4 h or more and between 2 and 3 h compared to those who reported 1 h or less. This finding is similar to that found for gambling behavior (24), alcohol use (57), and video game playing (23, 46).

Thus, this study provides the first application of the MCP to the assessment of the relative reinforcement value of social media networks time compared to a utilitarian reinforcer, such as a monetary reward. In this sense, the findings on reinforcer magnitude and delay effects are consistent with previous research that have applied behavioral economics to the study of non-substance–related addictions, such as problem gambling (58, 59) and obesity (60, 61).

One interesting aspect for mental health policy is comparing what Dixon (58) has done previously on problem behavior such as gambling in terms of the application of interventions based on targeted behavioral therapies such as acceptance and commitment therapy or cognitive behavioral therapy, which have shown positive results in populations of video game players (46). However, in the case of social media networks, it has been found in countries such as Spain, Chile, and Colombia, based on a sample of young adults and adolescents, that a significant percentage do not consider themselves to be dependent on these platforms, let alone addicted to them (62). This may be an effect of the normalization of the use of social media networks. Given their omnipresence in daily activities, which gives them a character of social acceptance, it becomes difficult to recognize that there may be an impact on quality of life or psychological and emotional wellbeing, particularly in the youngest users.

Nevertheless, there is evidence in the literature in favor of another alternative intervention, namely contingency management, which uses monetary incentives to influence individual behavior by making them prefer these incentives to the use of substances such as cocaine and alcohol (52, 63). In cooperation with the private sector, it may be possible to design mobile applications that provide coupons or discounts on brands of their choice in exchange for a reduction in daily social media usage. These applications can be tested on young users. Of course, technical and data privacy aspects must be considered, given the most recent EU regulations, for example. On the other hand, the fact that no differences were found in terms of gender usage can be interpreted that the use of social networks is so prevalent that there is no difference in their relative reinforcement value between men and women. This is a clear difference with video game playing, where there is a greater use of and dependence on video games in the case of men (24, 46).

Finally, this study provides a first approach to the use of the MCP in the context of social network use, as well as empirical evidence for the application of the BPM to digital consumption behavior in young users in conjunction with a methodology based on behavioral economics. Also, the present study aims to contribute to the growing literature on informational reinforcement (IR) in economic research. These findings pave the way for the search for possible clinical and social interventions in the face of this growing consumption phenomenon.

## 5. Limitations and future research

Some limitations must be acknowledged. First, placebo control conditions were not employed. Second, the sample was composed of college students; thus, the results should be generalized with caution. Future studies should include a more diverse sample, and, ideally, populations from different regions of the world could be compared because there are different rates of social network use in developed vs. emerging countries (56). Third, although six versions of the MCP were designed and applied, which corresponds to the same number used in previous studies that considered behavioral addictions such as gambling or video game playing (20, 23, 24), the same number of discrete choices was not applied as in the aforementioned studies. However, the literature does not establish yet a minimum or optimal number of choices within each version of the MCP. It is suggested that future research could address this inherent aspect of the MCP's measurement procedure. On the other hand, our results did not support the interaction effect of magnitude and delay of the alternative reinforcers. Thus, future research may consider replicating this research in the context of a mobile application intervention in a natural environment, which would provide ecological validity that complements the studies that have so far been carried out in experimental settings. By expanding the context to natural settings, that is, outside laboratory or experimental conditions, the MCP can be established as a methodology based on behavioral economics that contributes to the intervention of these emerging behaviors of dependence on social media networks content.

## Data availability statement

The raw data supporting the conclusions of this article will be made available by the authors, without undue reservation.

## Ethics statement

The studies involving human participants were reviewed and approved by Research Ethics Committee of the Rosario University Social Sciences Section. The patients/participants provided their written informed consent to participate in this study.

## Author contributions

All authors listed have made a substantial, direct, and intellectual contribution to the work and approved it for publication.

## References

[1] Bishop M. Consumer Informatics and Digital Health: Solutions for Health and Health Care. In:Edmunds N Hass C Holve E, editors. Consumer Informatics and Digital Health: Solutions for Health and Health Care. Switzerland: Springer (2019). p. 61–86.

[2] Kizgin H Jamal A Dey BL Rana NP. The impact of social media on consumers' acculturation and purchase intentions. Inf Syst Front. (2018) 20:503–14. 10.1007/s10796-017-9817-4

[3] Chaffey D. Global social media statistics research summary. Smart Insights Social Med Market. (2022) 272–325. 10.4324/9781003009498-6

[4] Lua A. 20 Top Social Media Sites to Consider For Your Brand in 2022. London: Routledge (2022).

[5] Abi-Jaoude E Naylor KT Pignatiello A. Smartphones, social media use and youth mental health. CMAJ. (2020) 192:E136–41. 10.1503/cmaj.190434 32041697 PMC7012622

[6] Abbasi IS. Social media addiction in romantic relationships: does user's age influence vulnerability to social media infidelity? Pers Individ Dif. (2019) 139:277–80. 10.1016/j.paid.2018.10.038

[7] Aschbrenner KA Naslund JA Tomlinson EF Kinney A Pratt SI Brunette MF. Adolescents' use of digital technologies and preferences for mobile health coaching in public mental health settings. Front Public Health. (2019) 7:1–9. 10.3389/fpubh.2019.00178 31312629 PMC6614191

[8] Dalvi-Esfahani M Niknafs A Alaedini Z Barati Ahmadabadi H Kuss DJ Ramayah T. Social media addiction and empathy: moderating impact of personality traits among high school students. Telemat Informatics. (2021) 57:101516. 10.1016/j.tele.2020.101516

[9] Lachmann B Sindermann C Sariyska RY Luo R Melchers MC Becker B. The role of empathy and life satisfaction in internet and smartphone use disorder. Front Psychol. (2018) 9:398. 10.3389/fpsyg.2018.00398 29636714 PMC5881138

[10] Sahin C. The predictive level of social media addiction for life satisfaction: a study on university students. Turkish Online J Educ Technol. (2017) 2017:515–20.

[11] Błachnio A Przepiorka A Pantic I. Association between Facebook addiction, self-esteem and life satisfaction: a cross-sectional study. Comput Human Behav. (2016) 55:701–5. 10.1016/j.chb.2015.10.026

[12] Levenson JC Shensa A Sidani JE Colditz JB Primack BA. The association between social media use and sleep disturbance among young adults. Prev Med. (2016) 85:36–41. 10.1016/j.ypmed.2016.01.001 26791323 PMC4857587

[13] Holland G Tiggemann M A. systematic review of the impact of the use of social networking sites on body image and disordered eating outcomes. Body Image. (2016) 17:100–10. 10.1016/j.bodyim.2016.02.008 26995158

[14] Samaha M Hawi NS. Relationships among smartphone addiction, stress, academic performance, and satisfaction with life. Comput Human Behav. (2016) 57:321–5. 10.1016/j.chb.2015.12.045

[15] Acuff SF MacKillop J Murphy JG. Applying behavioral economic theory to problematic Internet use: an initial investigation. Psychol Addict Behav. (2018) 32:846–57. 10.1037/adb0000404 30451521 PMC6247424

[16] Robayo-Pinzon O Foxall GR Montoya-Restrepo LA Rojas-Berrio S. Does excessive use of smartphones and apps make us more impulsive? An approach from behavioural economics. Heliyon. (2021) 7:e06104. 10.1016/j.heliyon.2021.e06104 33644439 PMC7887400

[17] Peng Y Zhou H Zhang B Mao H Hu R Jiang H. Perceived stress and mobile phone addiction among college students during the 2019 coronavirus disease: the mediating roles of rumination and the moderating role of self-control. Pers Individ Dif. (2022) 185:111222. 10.1016/j.paid.2021.111222 34429562 PMC8376708

[18] West R Ash C Dapore A Kirby B Malley K Zhu S. Problematic smartphone use: the role of reward processing, depressive symptoms and self-control. Addict Behav. (2021) 122:107015. 10.1016/j.addbeh.2021.107015 34146798

[19] Bickel WK Johnson MW Koffarnus MN MacKillop J Murphy JG. The behavioral economics of substance use disorders: reinforcement pathologies and their repair. Annu Rev Clin Psychol. (2014) 10:641–77. 10.1146/annurev-clinpsy-032813-153724 24679180 PMC4501268

[20] Griffiths RR Troisi JR Silverman K Mumford GK. Multiple-choice procedure: an efficient approach for investigating drug reinforcement in humans. Behav Pharmacol. (1993) 4:3–13. 10.1097/00008877-199302000-00001 11224166

[21] Bickel WK Marsch LA. Toward a behavioral economic understanding of drug dependence: delay discounting processes. Addiction. (2001) 96:73–86. 10.1046/j.1360-0443.2001.961736.x 11177521

[22] Higgins ST Heil SH Lussier JP. Clinical implications of reinforcement as a determinant of substance use disorders. Annu Rev Psychol. (2004) 55:431–61. 10.1146/annurev.psych.55.090902.142033 14744222

[23] Bassett DT Irons JG Schultz NR Correia CJ. Initial validation of the multiple-choice procedure for measuring video game playing. Addict Res Theory. (2020) 28:314–20. 10.1080/16066359.2019.1650350

[24] Butler LH Irons JG Bassett DT Correia CJ. Using the Multiple-Choice Procedure to Measure the Relative Reinforcing Efficacy of Gambling: Initial Validity Evidence Among College Students. J Gambl Stud. (2018) 34:513–20. 10.1007/s10899-017-9716-0 28932934

[25] Greenwald MK Stitzer ML. Antinociceptive, subjective and behavioral effects of smoked marijuana in humans. Drug Alcohol Depend. (2000) 59:261–75. 10.1016/S0376-8716(99)00128-3 10812286

[26] Benson TA Little CS Henslee AM Correia CJ. Effects of reinforcer magnitude and alternative reinforcer delay on preference for alcohol during a multiple-choice procedure. Drug Alcohol Depend. (2009) 100:161–3. 10.1016/j.drugalcdep.2008.09.005 19013028

[27] Jones HE Garrett BE Griffiths RR. Subjective and physiological effects of intravenous nicotine and cocaine in cigarette smoking cocaine abusers. J Pharmacol Exp Ther. (1999) 288:188–97. 9862770

[28] Madden GJ Petry NM Badger GJ Bickel WK. Impulsive and self-control choices in opioid-dependent patients and non-drug-using control participants: drug and monetary rewards. Exp Clin Psychopharmacol. (1997) 5:256–62. 10.1037/1064-1297.5.3.256 9260073

[29] Griffiths RR Rush CR Puhala KA. Validation of the multiple-choice procedure for investigating drug reinforcement in humans. Exp Clin Psychopharmacol. (1996) 4:97–106. 10.1037/1064-1297.4.1.9719013028

[30] Amlung M Mackillop J. Understanding the effects of stress and alcohol cues on motivation for alcohol via behavioral economics. Alcohol Clin Exp Res. (2014) 38:1780–9. 10.1111/acer.12423 24890323 PMC4049358

[31] Madden GJ Johnson PS. A delay-discounting primer. In:Madden GJ Bickel WK, editors. Impulsivity: The Behavioral and Neurological Science of Discounting. Washington, DC: American Psychological Association (2010). p. 11–37.

[32] Yi R de la Piedad X Bickel WK. The combined effects of delay and probability in discounting. Behav Processes. (2006) 73:149–55. 10.1016/j.beproc.2006.05.001 16759821

[33] Foxall GR Oliveira-Castro JM James VK Schrezenmaier TC. The Behavioral Economics of Brand Choice. (2007). p. 1–292. 10.1057/9780230596733_1

[34] Ballard IC Kim B Liatsis A Aydogan G Cohen JD McClure SM. More is meaningful: the magnitude effect in intertemporal choice depends on self-control. Psychol Sci. (2017) 28:1443–54. 10.1177/0956797617711455 28858559 PMC5959284

[35] Macedo I Fernandes C Barbosa F Marques-Teixeira J. Delay discounting in aging: the influence of cognitive and psychological variables. Behav Neurosci. (2022) 136:392–403. 10.1037/bne0000518 35604715

[36] Gershman SJ Bhui R. Rationally inattentive intertemporal choice. Nat Commun. (2020) 11:3365. 10.1038/s41467-020-16852-y 32620804 PMC7335105

[37] Thaler R. Some empirical evidence on dynamic inconsistency. Econ Lett. (1981) 8:201–7. 10.1016/0165-1765(81)90067-7

[38] Loewenstein G Thaler RH. Anomalies: intertemporal choice. J Econ Perspect. (1989) 3:181–93. 10.1257/jep.3.4.181

[39] Prelec D Loewenstein G. Decision making over time and under uncertainty: a common approach. Manage Sci. (1991) 37:770–86. 10.1287/mnsc.37.7.770

[40] Arfer KB Luhmann CC. The predictive accuracy of intertemporal-choice models. Br J Math Stat Psychol. (2015) 68:326–41. 10.1111/bmsp.12049 25773127

[41] Foxall GR Sigurdsson V. When loss rewards: the near-miss effect in slot machine gambling. Anal Gambl Behav. (2012) 6:5–22.26018845

[42] Foxall GR. Radical behaviorist interpretation: generating and evaluating an account of consumer behavior. Behav Anal. (1998) 21:321–54. 10.1007/BF03391971 22478315 PMC2731411

[43] Foxall GR. Invitation to consumer behavior analysis. J Organ Behav Manage. (2010) 30:92–109. 10.1080/01608061003756307

[44] Foxall GR. Advanced Introduction to Consumer Behavior Analysis. Cheltenham, Glos. and Northampton, MA: Edward Elgar Publishing Ltd. (2018). p. 160.

[45] Foxall GR. Addiction as Consumer Choice: Exploring the Cognitive Dimension. First Edit. New York: Routledge Taylor and Francis Group (2016). p. 222. 10.4324/9780203794876

[46] Buono FD Sprong ME Lloyd DP Cutter CJ Printz DMB Sullivan RM. Delay discounting of video game players: comparison of time duration among gamers. Cyberpsychology, Behav Soc Netw. (2017) 20:104–8. 10.1089/cyber.2016.0451 28118044 PMC5312545

[47] Gilroy SP Picardo R. Applications of operant demand to treatment selection III: consumer behavior analysis of treatment choice. J Exp Anal Behav. (2022) 118:46–58. 10.1002/jeab.758 35416300

[48] Gilroy SP Kaplan BA. Modeling treatment-related decision-making using applied behavioral economics: caregiver perspectives in temporally-extended behavioral treatments. J Abnorm Child Psychol. (2020) 48:607–18. 10.1007/s10802-020-00619-6 31982979

[49] Sun J Liu Q Yu S. Child neglect, psychological abuse and smartphone addiction among Chinese adolescents: the roles of emotional intelligence and coping style. Comput Human Behav. (2019) 90:74–83. 10.1016/j.chb.2018.08.032

[50] Billieux J. Problematic use of the mobile phone: a literature review and a pathways model. Curr Psychiatry Rev. (2012) 8:299–307. 10.2174/157340012803520522

[51] Barnes SJ Pressey AD Scornavacca E. Mobile ubiquity: understanding the relationship between cognitive absorption, smartphone addiction and social network services. Comput Human Behav. (2019) 90:246–58. 10.1016/j.chb.2018.09.013

[52] Krishnamurti T Ling Murtaugh K Van Nunen L Davis AL Ipser J Shoptaw S. Spending money to make change: association of methamphetamine abstinence and voucher spending among contingency management pilot participants in South Africa. J Subst Abuse Treat. (2020) 112:60–7. 10.1016/j.jsat.2020.01.014 32199547 PMC7089867

[53] Tutgun-ünal A Deniz L. Development of the Social Media Addiction Scale. AJIT-e Bilişim Teknol Online Derg. (2015) 6:51–70. 10.5824/1309-1581.2015.4.004.x36293756

[54] Cruz Rambaud S Muñoz Torrecillas MJ. An analysis of the anomalies in traditional discounting models. Int J Psychol Psychol Therapy. (2004) 4:105–28.

[55] Kemp S,. Digital 2022: Colombia. (2022). Available online at: https://datareportal.com/reports/digital-2022-colombia (accessed April 15, 2022).

[56] Kemp S,. Digital 2022 Global Overview Report. (2022). Available online at: https://kepios.com/reports (accessed April 15, 2022).

[57] Correia CJ Little C. Use of a multiple-choice procedure with college student drinkers. Psychol Addict Behav. (2006) 20:445–52. 10.1037/0893-164X.20.4.445 17176179

[58] Dixon MR Jacobs EA Sanders S. Contextual control of delay discounting by pathological gamblers. J Appl Behav Anal. (2006) 39:413–22. 10.1901/jaba.2006.173-05 17236338 PMC1702333

[59] Alessi SM Petry NM. Pathological gambling severity is associated with impulsivity in a delay discounting procedure. Behav Processes. (2003) 64:345–54. 10.1016/S0376-6357(03)00150-5 14580703

[60] Rasmussen EB Lawyer SR Reilly W. Percent body fat is related to delay and probability discounting for food in humans. Behav Processes. (2010) 83:23–30. 10.1016/j.beproc.2009.09.001 19744547

[61] Weller RE Cook III EW Avsar KB Cox JE. Obese women show greater delay discounting than healthy-weight women. Appetite. (2008) 51:563–9. 10.1016/j.appet.2008.04.010 18513828

[62] Almenara JC Pérez SM Ortiz R V Nuñez JPL Hernandez MLO López IH. Students addiction to online social networks: a study in the Latin American context. Rev Complut Educ. (2020) 31:1–12. 10.5209/rced.61722

[63] Petry NM Alessi SM Ledgerwood DM. Contingency management delivered by community therapists in outpatient settings. Drug Alcohol Depend. (2012) 122:86–92. 10.1016/j.drugalcdep.2011.09.015 21981991 PMC3290694

