# Supplementary material for: Social media sites users' choice between utilitarian and informational reinforcers assessed using temporal discounting
Source: Front Public Health. 2023 Feb 10;11:960321. doi: 10.3389/fpubh.2023.960321 (PMC9950497; doi:10.3389/fpubh.2023.960321)
Supplement: Supplementary file 1 [file Table_1.docx]

**Appendix**

**Study on the use of social media networks and alternative incentives**

Dear participant,

We would like to send you a warm greeting. Thank you very much for your participation.

The purpose of this research is to "establish the relative value of the use of social media networks with respect to possible alternative incentives". In that sense, the information provided in this form is confidential and exclusively for the use of this research. Your data will be processed and analysed in an aggregated and anonymous way, and will only be used for research purposes, therefore your personal data will not be published or shared in any media, in compliance with the data processing and habeas data protection policy.

Duration: approximately 20 minutes.

If you have any questions or concerns, we will be happy to answer them by email: [oscar.robayo@urosario.edu.co](mailto:oscar.robayo@urosario.edu.co)

1. **Demographic questions**
   1. Age in years completed (only enter numbers): _________
   2. What gender do you identify with?

Woman

Man

Other

- 1. What level of education have you attained or are you currently pursuing?

Technician - Professional Technician

Technologist

Professional - Undergraduate

Specialization - Technological Specialization

Master's Degree

Doctorate

Postdoctoral

- 1. Please tick all boxes that define your current employment status:

Part-time employment

Full-time employment

Unemployed

Self-employed

Student

Pensioner

e. What is your socio-economic status?

1. Low-low

2. Low

3. Lower-middle

4. Medium

5. Medium-high

6. High

f. What is your personal income range? (Values are in current legal minimum wages and in Colombian pesos)

Less than 1 SMMLV (908,526)

Between 1 SMMLV (908,526) and less than 3 SMMLV (2,725,578)

Between 3 SMMLV (2,725,578) and less than 5 SMMLV (4,542,630)

More than 5 SMMLV (4,542,630) and less than 7 SMMLV (6,359,682)

More than 7 SMMLV (6,359,682)

II. **Usage of social media networks**

1. Which social media network do you use most of the time per day? (Does not include instant messaging applications: WhatsApp, Telegram, etc.).

Facebook

Instagram

TikTok

YouTube

Twitter

Pinterest

Snapchat

LinkedIn

Other: _____________

b. Approximately how much time on average per day do you use the social network you indicated in the previous question?

1 hour or less

Between 1 and 2 hours

Between 2 and 3 hours

4 hours or more

c. At what time of the day do you consider that you use the social network you indicated above the most? (You can select more than one option):

Morning

Afternoon

Evening

III. **Choice questions**

Imagine the following hypothetical situation. You have been invited to participate in a laboratory study and you will be in the university laboratory for 180 minutes (3 hours). During that time, you will be completing activities such as crossword puzzles, word searches, etc. for extra credit at the university. You will not have access to your mobile phone or any other means of communication. However, 15 minutes into the session, the researcher tells you that you can choose between earning some money or having access to your social networks (as many as you want to use) for the next 5, 15, or 45 minutes. The researcher shows you your smartphone and then asks you to make a series of choices, which are listed below.

For each question, you must indicate your preference by selecting one of the two available options. You must answer all questions. Please note that regardless of your choice, you will remain in the lab for the 180 minutes (3 hours) to earn the extra credit at the university. These options are hypothetical, but please answer as if you were actually in the situation.

**Situation 1**

You will then be asked to answer several questions in which you will choose between using your social networks (as many as you want) for 5 minutes immediately or receiving a certain amount of money (Colombian pesos) immediately.

1.1 Which alternative do you prefer?

A. 5 minutes of use of your social networks browsing immediately.

B. Receive $10000 immediately.

1.2 Which alternative do you prefer?

A. 5 minutes of use of your social networks by browsing immediately.

B. Receive $12000 immediately.

1.3 Which alternative would you prefer?

A. 5 minutes of use of your social networks by browsing immediately.

B. Receive $15000 immediately.

1.4 Which alternative would you prefer?

A. 5 minutes of use of your social networks by browsing immediately.

B. Receive $20000 immediately.

1.5 Which alternative would you prefer?

A. 5 minutes of use of your social networks by browsing immediately.

B. Receive $25000 immediately.

**Situation 2**

You will then have to answer several questions in which you will choose between using your social networks (as many as you want) for 5 minutes immediately or waiting for a period of one week to receive a certain amount of money (Colombian pesos).

2.1 Which alternative do you prefer?

A. 5 minutes of use of your social networks browsing immediately.

B. Receive $10000 within a week.

2.2 Which alternative do you prefer?

A. 5 minutes of use of your social networks by browsing immediately.

B. Receive $12000 within a week.

2.3 Which alternative do you prefer?

A. 5 minutes of use of your social networks by browsing immediately.

B. Receive $15000 within a week.

2.4 Which alternative do you prefer?

A. 5 minutes of use of your social networks by browsing immediately.

B. Receive $20000 within a week.

2.5 Which alternative would you prefer?

A. 5 minutes of social media use by browsing immediately.

B. Receive $25000 within a week.

**Situation 3**

You will then have to answer several questions in which you will choose between using your social networks (as many as you want) for 15 minutes immediately or receiving a certain amount of money (Colombian pesos) immediately.

3.1 Which alternative do you prefer?

A. 15 minutes of use of your social networks browsing immediately.

B. Receive $10000 immediately.

3.2 Which alternative do you prefer?

A. 15 minutes of use of your social networks by browsing immediately.

B. Receive $12000 immediately.

3.3 Which alternative would you prefer?

A. 15 minutes of use of your social networks by browsing immediately.

B. Receive $15000 immediately.

3.4 Which alternative would you prefer?

A. 15 minutes of use of your social networks by browsing immediately.

B. Receive $20000 immediately.

3.5 Which alternative would you prefer?

A. 15 minutes of use of your social networks by browsing immediately.

B. Receive $25000 immediately.

**Situation 4**

You will then have to answer several questions in which you will choose between using your social networks (as many as you want) for 15 minutes immediately or waiting for a period of one week to receive a certain amount of money (Colombian pesos).

4.1 Which alternative do you prefer?

A. 15 minutes of use of your social networks by browsing immediately.

B. Receive $10000 within a week.

4.2 Which alternative do you prefer?

A. 15 minutes of use of your social networks by browsing immediately.

B. Receive $12000 within a week.

4.3 Which alternative do you prefer?

A. 15 minutes of use of your social networks by browsing immediately.

B. Receive $15000 within a week.

4.4 Which alternative do you prefer?

A. 15 minutes of social media use by browsing immediately.

B. Receive $20000 within a week.

4.5 Which alternative would you prefer?

A. 15 minutes of social media use by browsing immediately.

B. Receive $25000 within a week.

**Situation 5**

You will then have to answer several questions in which you will choose between using your social networks (as many as you want) for 45 minutes immediately or receiving a certain amount of money (Colombian pesos) immediately.

5.1 Which alternative do you prefer?

A. 45 minutes of use of your social networks browsing immediately.

B. Receive $10000 immediately.

5.2 Which alternative do you prefer?

A. 45 minutes of use of your social networks by browsing immediately.

B. Receive $12000 immediately.

5.3 Which alternative would you prefer?

A. 45 minutes of use of your social networks by browsing immediately.

B. Receive $15000 immediately.

5.4 Which alternative would you prefer?

A. 45 minutes of social media use by browsing immediately.

B. Receive $20000 immediately.

5.5 Which alternative would you prefer?

A. 45 minutes of use of your social networking sites by browsing immediately.

B. Receive $25000 immediately.

**Situation 6**

You will then have to answer several questions in which you will choose between using your social networks (as many as you want) for 45 minutes immediately or waiting for a period of one week to receive a certain amount of money (Colombian pesos).

6.1 Which alternative do you prefer?

A. 45 minutes of use of your social networks by browsing immediately.

B. Receive $10000 within a week.

6.2 Which alternative do you prefer?

A. 45 minutes of use of your social networks by browsing immediately.

B. Receive $12000 within a week.

6.3 Which alternative do you prefer?

A. 45 minutes of use of your social networks by browsing immediately.

B. Receive $15000 within a week.

6.4 Which alternative do you prefer?

A. 45 minutes of social media use by browsing immediately.

B. Receive $20000 within a week.

6.5 Which alternative would you prefer?

A. 45 minutes of social media use by browsing immediately.

B. Receive $25000 within a week.

IV. **Social Media Addiction Scale (SMAS)**

For the following questions please consider the relationship you have with social networks in general. Please note that the response scale includes the following options: 1. Never, 2. Rarely, 3. Sometimes, 4. Often, and 5. Always.

1. I think about what must be happening now on my social networks.

2. The first thing I do in the day is to check my social networks.

3. When I don't check my social networks for a while, the thought of checking them occupies my mind.

4. I think my life would be boring, empty, and bland if I didn't have social media.

5. When I am not connected to the Internet, I think a lot about checking my social networks.

6. I wonder what is going on in my social networks now.

7. There are times when I spend more time on social networks than I think I do.

8. When I am using social networks and I want to do something else, I say to myself "a few more minutes".

9. I can't stop using social networks for a long time.

10. There are times when I use social networks more than I planned.

11. I don't notice how time goes by while using social networks.

12. I spend a lot of time on different activities (chatting, looking at pictures and posts, etc.) on social networks.

13. I use social networks to forget my personal problems.

14. I spend time on social networks when I feel lonely.

15. I prefer to surf social networks to free myself from negative thoughts about my life.

16. When I get bored with my problems, the best place to find refuge is social networks.

17. I forget about everything when I am using my social networks.

18. There are times when I try to stop using social networks and I don't succeed.

19. I strongly want to reduce the amount of time I use social networking sites.

20. I make useless efforts to stop using social networking sites.

21. I make futile efforts to decrease the time I spend on social networking sites.

22. I try to reduce the amount of time I spend on social networks, but I do not succeed.

23. I use social networking sites more, even if it negatively affects my work or studies.

24. I give less priority to my hobbies and leisure activities because of social networking.

25. There are times when I neglect my loved ones and friends because of using social networks.

26. There are times when I neglect my friends because of social networking.

27. Because of social networking, I can't finish my activities on time.

28. In order to spend more time on social networking, I neglect activities related to study or work.

29. I prefer to spend time on social networking sites rather than spending time with my friends.

30. My study or work is interrupted by the time I spend on social networks.

31. My productivity decreases because of my use of social networking sites.

32. I would rather spend time on social networking sites than hanging out with my friends.

33. People criticize me because of the time I spend on social networks.

34. I try to hide the time I spend on social networking sites from other people.

35. There are times when I forget to eat because of social networking.

36. There are times when I spend less time on self-care because of using social networks.

37. I have sleep disruptions/disturbances because of my use of social networks.

38. Sometimes I have physical problems (backaches, headaches, eye pains) due to the use of social networking.

39. The use of social networks causes problems in my relationships with people who are important to me.

40. The use of social networks causes problems in my life.

41. As the things I must do increase, my desire to use social networks increases at the same rate.

1. **Contact information**
   1. Name
   2. Mobile phone number
   3. Institutional e-mail
